# Sex Differences in Correlation with Gene Expression Levels between *Ifi200* Family Genes and Four Sets of Immune Disease-Relevant Genes

**DOI:** 10.1155/2018/1290814

**Published:** 2018-08-28

**Authors:** Yanhong Cao, Lishi Wang, Cong-Yi Wang, Jicheng Ye, Ying Wang, Tiantian Li, Franklin Garcia-Godoy, Dianjun Sun, Weikuan Gu, Arnold E. Postlethwaite

**Affiliations:** ^1^Center for Endemic Disease Control, Center for Disease Control and Prevention, Harbin Medical University, Harbin 150081, China; ^2^Departments of Orthopaedic Surgery-Campbell Clinic and Pathology, University of Tennessee Health Science Center (UTHSC), Memphis, TN 38163, USA; ^3^Department of Basic Medical Research, Inner Mongolia Medical University, Jinshan New Investment and Development Zones, Hohhort, Inner Mongolia 010110, China; ^4^The Center for Biomedical Research, Tongji Hospital, Tongji Medical College, Huazhong University of Science and Technology, 1095 Jiefang Ave, Wuhan 430030, China; ^5^Bioscience Research Center, College of Dentistry, University of Tennessee Health Science Center (UTHSC), 875 Union Avenue, Memphis, TN 38163, USA; ^6^Research Service, Memphis VA Medical Center, 1030 Jefferson Avenue, Memphis, TN 38104, USA; ^7^Division of Connective Tissue Diseases, Department of Medicine, University of Tennessee Health Science Center (UTHSC), Memphis, TN 38163, USA

## Abstract

**Background:**

The HIN-200 family genes in humans have been linked to several autoimmune diseases—particularly to systemic lupus erythematosus (SLE) and rheumatoid arthritis (RA). Recently, its human counterpart gene cluster, the *Ifi200* family in mice, has been linked to spontaneous arthritis disease (SAD). However, many immune-mediated diseases (including RA and SLE) show gender difference. Understanding whether or not and how these genes play a role in sex difference in immune-mediated diseases is essential for diagnosis/treatment.

**Methods:**

This study takes advantage of the whole genome gene expression profiles of recombinant inbred (RI) strain populations from female and male mice to analyze potential sex differences in a variety of genes in disease pathways. Expression levels and regulatory QTL of *Ifi200* family genes between female and male mice were first examined in a large mouse population, including RI strains derived from C57BL/6J, DBA/2J (BXD), and classic inbred strains. Sex similarities and differences were then analyzed for correlations with gene expression levels between genes in the *Ifi200* family and four selected gene sets: known immune *Ifi200* pathway-related genes, lupus-relevant genes, osteoarthritis- (OA-) and RA-relevant genes, and sex hormone-related genes.

**Results:**

The expression level of *Ifi202b* showed the most sex difference in correlation with known immune-related genes (the *P* value for *Ifi202b* is 0.0004). *Ifi202b* also showed gender difference in correlation with selected sex hormone genes, with a *P* value of 0.0243. When comparing coexpression levels between *Ifi200* genes and lupus-relevant genes, *Ifi203* and *Ifi205* showed significant sex difference (*P* values: 0.0303 and 0.002, resp.). Furthermore, several key genes (e.g., *Csf1r*, *Ifnb1*, *IL-20*, *IL-22*, *IL-24*, *Jhdm1d*, *Csf1r*, *Ifnb1*, *IL-20*, *IL-22*, *IL-24*, and *Tgfb2* that regulate sex differences in immune diseases) were discovered.

**Conclusions:**

Different genes in the *Ifi200* family play different roles in sex difference among dissimilar pathways of these four gene groups.

## 1. Background

Recently, *Ifi200* genes, including the human HIN-200 gene cluster and its mouse counterpart, the interferon inducible-200 (*Ifi200*) family, have been linked to several autoimmune diseases [1–5]. These genes have been linked to SLE. As early as in 1994, IFI16 was recognized as a target of antinuclear antibodies in patients with SLE [6]. The Ifi200 family genes as modifiers for the SLE susceptibility have been nicely summarized in a review by Choubey [7]. A recent study suggests that anti-IFI16 antibodies hold the potential to serve as a new biomarker of disease activity in SLE [8]. IFI16 is in the same paralogue with pyrin and is an HIN domain family member 1 (PYHIN1 or IFIX), myeloid cell nuclear differentiation antigen (MNDA), and absent in melanoma 2 (AIM2) on human chromosome 1 as a human HIN-200 gene cluster. Similar function was found for other genes in this cluster [4, 6]. AIM2 was found to facilitate apoptotic DNA-induced SLE via arbitrating macrophage functional maturation [9]. These genes are also linked to RA. In particular, circulating IFI16 has been found to correlate with clinical and serological features in RA [1]. In their report, Alunno et al. showed that high levels of circulating IFI16 in RA are more frequent in RF/anti-CCP-positive RA patients and significantly associated with pulmonary involvement. Most recently, a study using the IL-1RA-deficient mouse model found that decreased expression levels of Ifi genes were associated with increased resistance to SAD [10]. In addition, previously, interferon-inducible protein-10 (IP-10) was found to be linked to RA, although it is not located in the human HIN-200 cluster [11, 12]. These findings suggest that it is possible that *Ifi200* family genes may play an important role in the development of arthritis.

Both SLE and RA have increased prevalence in women compared to men. Whether the function of *Ifi200* genes shows gender (or sex) difference is not completely understood. A few studies have been performed using animal models. Panchanathan et al. reported that cell type- and gender-dependent factors differentially regulate the expression of the AIM2 and p202 proteins—thus, suggesting opposing roles for these two proteins in innate immune responses in SLE [13]. Yang et al. reported the sex-dependent differential activation of NLRP3 and AIM2 inflammasomes in SLE macrophages [14]. Regulation of *Ifi202* has also been linked to sex hormones [15]. Their studies suggest that there is potentially sex difference in the function of *Ifi200* genes. Therefore, understanding whether there is a sex difference and how their expression and function show sex difference would represent a significant advance in the elucidation of molecular mechanism(s) of *Ifi200* genes in autoimmune diseases.

Its orthologue cluster in mouse is *Ifi200* on mouse chromosome 1. In mice, the *Ifi200* family genes *Ifi202b*, *Ifi203*, *Ifi204*, *Ifi205*, and *Mnda* are known to reside on chromosome 1. This family gene cluster is between 173,747,293 (*Ifi204*) bp and 174,031,810 (*Ifi205*) bp. The *Ifi200* family genes are next to each other and are called the interferon-inducible (Ifi) gene 200 cluster. How these genes interact and exert influence on each other is not clear. Thus, elucidation of interaction mechanisms among genes in the *Ifi200* cluster might enhance our understanding of relationships between these genes and autoimmune diseases, in particular the RA. However, due to the requirements for appropriate sample collections (e.g., both sexes at the same age, unified genomic background, and controlled environment), such a study using human populations has been difficult.

Animal models have been widely used to study topics that could not be easily studied using human populations. In particular, rodent models such as those in mice have contributed tremendously to our understanding of human genetics and genomics. We will examine the sex similarity and difference using data of whole genome gene expression profiles from a well-known mouse population of recombinant inbred (RI) strains derived from C57BL/6J and DBA/2J (BXD), which is the largest RI mouse population and with remarkable data on whole genome expression profiles and phenotypes [16–18]. The first set of 36 BXD RI strains was originally established in 1930s at The Jackson Laboratory [19]. Over the last more than a half century, the BXD RI strains have expanded into a population with almost of a hundred RI strains. Among rodent animal models, this is the largest animal RI strains in history [20]. Unlike F2 population, one RI strain needs intercrossing within the strain more than 20 generations before it established its homozygous status and survival of inbred selection. In the last decade, BXD RI strains have been used widely as the only reliable RI strain population. As of May, 2018, PubMed posted more than 500 publications that their research is based on the BXD strains. The analysis tools for these RI strains are provided by GeneNetwork [21]. These tools have been tested, applied, and approved for the last decade [22].

## 2. Materials and Methods

### 2.1. Mouse Gene Expression Data Sets

For study of the sex difference and similarity of gene expression profiles, we used the whole gene expression profiles of spleen from female and male mice in a population of recombinant inbred (RI) strains from BXD (derived from C57BL/6J and DBA/2J) [18].

The data sets, UTHSC Affy MoGene 1.0 ST Spleen (Dec10), were from GeneNetwork (http://www.genenetwork.org/webqtl/main.py). There were separate sets of whole genome expression profiles from the spleen of female and male mice. In both male and female mouse sets, the whole genome gene expression profiles were all from a total of 85 strains, including 64 BXD strains, parental strain C57BL/6J, two reciprocal F1 hybrids (B6D2F1 and D2B6F1), and 18 other common inbred strains. The spleen was profiled using the Affymetrix GeneChip Mouse Gene 1.0 ST array. In most cases, two arrays were processed per strain—one for males and one for females [23].

### 2.2. EQTL Mapping

We followed the standard protocol provided by GeneNetwork in conducting the eQTL mapping of female and male eQTL from spleen. eQTL maps were generated by using the command “Interval” of “Mapping Tools” at GeneNetwork. Permutation tests of 3000 was used to map the eQTLs to confirm accuracy of eQTL mapping. A simple regression method was used by GeneNetwork for mapping the expression QTL of genes with flanking markers [24]. The expression values from different strains were considered as phenotypes. Molecular markers along the chromosomes were used as genotypes or indicators of locations on chromosomes. The expression values were then compared for the probability of a specific genotype at a test location between two flanking markers. Significance of eQTL at a location was evaluated with statistical probabilities to eventually generate eQTL.

### 2.3. Gene Network Construction

Gene networks were constructed for genes in the *Ifi200* pathway and for sex hormones. The graphic application tools in GeneNetwork were used for the gene network construction. Spring Model layout (force reduction) was selected as the graphic method for all graphic subjects. The criteria for the strong correlation, correlation, and no correlation were the absolute value of *R* equal to or >0.7 (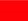 red color for positive and 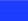 blue color for negative correlation), between 0.36 and 0.69 (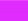 pink color for positive and 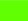 green color for negative correlation), and between 0 and 0.35, respectively (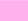 light pink color for positive and 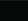 black color for negative correlation) [25]. In case of multiple probes, all of the probes were initially employed in the construction of gene network. The probe with the highest expression level was chosen from highly positive related probes of the same gene for the final construction of the gene network. For construction of graphic gene network, unless specified, we default to show the Pearson correlation coefficients > 0.35 or ≤0.35 between genes. The graph's canvas is 40.0 cm by 40.0 cm. The node labels and edge labels are drawn with a 16.0-point font.

### 2.4. Gene Categories Analyzed for Association with Genes in the *Ifi200* Family

Genes collected from four categories were used in the analysis for correlations of their expression levels to the expression levels of the *Ifi200* family. We first included genes that are potentially connected to *Ifi200* genes and well known for their importance in the immune system. These genes included *FoxP3*, *Tgfb*, type I IFN, and translocated promoter region (Tpr) protein. Type I IFNs belong to the class II family of *α*-helical cytokines, which includes type II *IFN-γ*, *IL-17*, the newly identified IFN-*λ*s, *IL-10*, and several IL-10 homologs (*IL-19*, *IL-20*, *IL-22*, *IL-24*, and *IL-26*) [26–29]. We also included potential upstream genes such as *Csf1r* [30], *Gata4*, *Nkx2.5*, and *Tbx5* [31].

We next assessed genes with different expression levels in patients with SLE or RA and animal models of SLE, RA, and OA. These genes include *Ets-1* and *FoxP3* [32]; *ITGAM* and Fc*γ*RIIIA [33]; *PD-1.3A*, *C4AQ0*, and *MBL* [34]; *AlFadhli* (*IRF9*, *ABCA1*, *APOBEC3*, *CEACAM3*, *OSCAR*, *TNFA1P6*, *MMP9*, and *SLC4A1*) [35]; *FCGR3A* and *FCGR3B* [36]; *Tlr7* [37]; *TBX21* and *IFNG* [38]; *CD95* and *CCR7* [39]; Fkbp11 [40]; *JHDM1D* and *HDAC1-3* [41]; *IL-28RA* [42]; and *pSTAT1* and *ETS1* [43]. For genes associated with arthritis, we used genes expressed in RA or OA [44–46]. These genes are involved in immune response (CD97, FYB, CXCL1, IKBKE, and CCR1), inflammatory response (*CD97*, *CXCL1*, *C3AR1*, *CCR1*, and *LYZ*), homeostasis (*C3AR1*, *CCR1*, *PLN*, *CCL19*, and *PPT1*), and other processes (*JAK/STAT*, *SOCS*, *c-IAP1*, *c-IAP2*, *XIAP*, *PI3K/Akt/mTOR*, *SAPK/MAPK*, and *IL-20-*induced *TNF-α*, *IL-1β*, *MMP-1*, and *MMP-*13) [44, 46].

Probes for sex hormones were searched in GeneNetwork from the whole genome expression profiles of the spleen of female and male mice by using the key words “estradiol,” “progesterone,” and “testosterone.” The expression levels of these genes were correlated with expression levels of genes in the *Ifi200* family.

### 2.5. Data Organization and Comparison

The following symbols are used in the data analysis and organization: *R* is the correlation between each gene in the *Ifi200* family and each gene from gene sets of different categories. *R* values were obtained from matrix analysis and graphic analysis at GeneNetwork [16, 47]; *R* is the correlation between different *R* values of different comparisons; *P* is the result of *t*-test; and Raa is the average of the absolute *R* value, which is calculated by Raa = ∑Ri/*n*, where Ri is the absolute *R* value between a gene in a gene group and *n* is the total number of *R* values.

When analyzing the correlations between Ifi family genes and each set of other selected genes, the collective *R* values between each *Ifi200* gene to the genes in each category are treated as a set. The four sets of *R* values, for example, *Ifi202b*, *Ifi203*, *Ifi204*, and *Ifi205* in each category (known relevant immune and *Ifi200* pathways, lupus, arthritis, and sex hormones) in female are compared to the set in male. The *P* values from *t*-tests and *R* values from correlation tests are used as criteria to judge their similarities and differences. The relevance of expression levels between a gene group and *Ifi200* genes was evaluated by the average of absolute *R* values between genes in a group and *Ifi200* genes. Raa is used to compare the strength of association of the *Ifi200* family genes to genes in each category.

### 2.6. Statistical Analysis

Student's *t*-test was used for the comparison of samples. The criteria for statistical significance follow the standard values. Thus, *P* ≤ 0.01 and *P* ≤ 0.05 represent strong significant difference and difference, respectively. In the construction of network graphs, the *R* absolute value > 0.50 was considered as the indication of the threshold for the real connection line between two genes or probes.

## 3. Results

### 3.1. The Expression Levels in Female and Male Mice of Genes of the *Ifi200* Family

**Figure 1:**
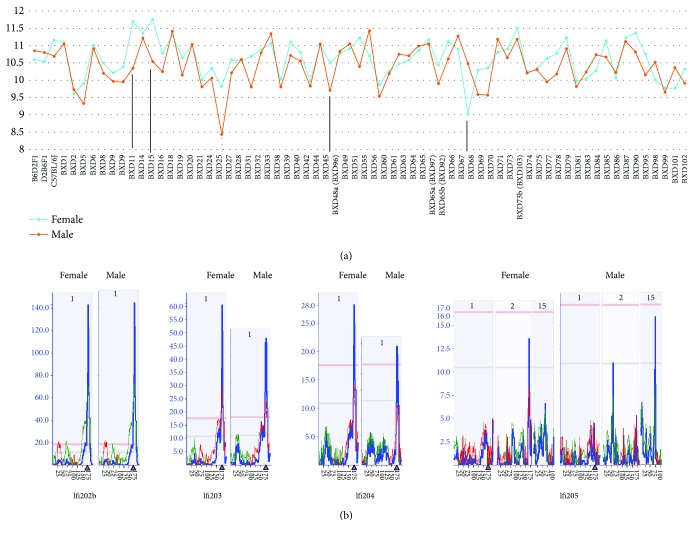
Information of expression levels of *Ifi200* genes in female and male mice among mouse strains. (a) Expression level of *Ifi202b* between female and male in different mouse strains. The numbers on the vertical bar indicate the relative scale of the gene expression level. On horizontal bar are the names of mouse strains. Strains with most sex difference are indicated by black bars. (b) Locations of eQTL that regulate the expression levels of genes of *Ifi200*. The figure contains four groups of pictures based on four *Ifi200* genes (*Ifi2002b*, *Ifi203*, *Ifi204*, and *Ifi205*). On the left of each group of pictures is the LRS (likelihood ratio statistic), which measures the association of linkage between the expression levels of *Ifi200* family genes and particular genotype markers on mouse chromosomes. Gene names and sex are listed at the bottom and top of each picture, respectively. The number of each column of mapping picture is the number of chromosome where the eQTL is located. The two bars, the pink and grey ones, are the threshold levels for significant and suggestive levels of an eQTL.

The expression levels of genes of the *Ifi200* family (*Ifi202b*, *Ifi203*, *Ifi204*, *Ifi205*, and *Mnda*) were examined. One probe for each of the *Ifi202b*, *Ifi203*, *Ifi204*, and *Ifi205* genes was identified from female and male populations except *Mnda.* The probe for beta-actin was used as the control. The *R* value of beta-actin between female and male mice was 0.166341. The *R* values for *Ifi202b*, *Ifi203*, *Ifi204*, and *Ifi205* were 0.9502, 0.6332, 0.6712, and 0.3960, respectively. The *t*-test resulted in a *P* value of 0.931. The *P* values between female and male for *Ifi202b*, *Ifi203*, *Ifi204*, and *Ifi205* were 0.857239, 0.003656, 0.155603, and 0.340488, respectively. Further examination of the expression levels of *Ifi203* revealed that while the expression level of *Ifi203* between female and male mice in most strains was similar, a few strains showed considerable difference such as strains BXD27 and BXD58 (Figure 1(a)).

eQTL mapping suggests that *Ifi202b*, *Ifi203*, and *Ifi204* in both sexes were all mapped on chromosome 1 (Figure 1(b)). However, the eQTL of *Ifi205* in female mice was mapped on chromosome 2, while in male mice, it was mapped on chromosome 15 (Figure 1(b)). This initial analysis suggests that there was potential sex difference among genes in the *Ifi200* family.

### 3.2. The Association between *Ifi200* Genes and Genes of Other Important Immune-Related Genes

**Figure 2:**
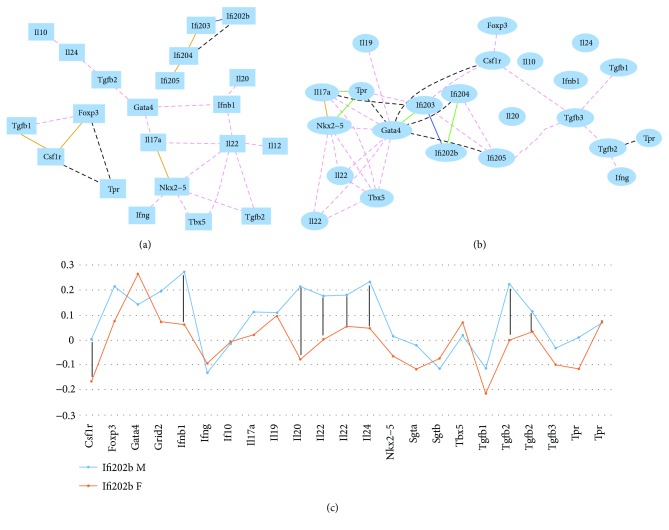
Gene network and sex difference of Ifi family genes with important immune-related genes in the spleen. (a) Gene network of Ifi family genes in male mice. The 21 nodes in the graph below show the selected traits. All nodes are displayed. The 25 edges between the nodes, filtered from the 210 total edges and drawn as curves, and the node labels are drawn with an 18.0-point font, and the edge labels are drawn with a 15.0-point font. (b) Gene network of Ifi family genes in female mice. The 23 nodes in the graph below show the selected traits. The 36 edges between the nodes, filtered from the 253 total edges and drawn as curves, and the node labels are drawn with a 16.0-point font, and the edge labels are drawn with a 16.0-point font. (c) Sex difference for correlation of expression levels between *Ifi202b* and important immune-related genes in the spleen. The numbers on the vertical bar indicate the scale of *R* values between the expression level of *Ifi202b* and each gene listed at the bottom of the figure. Female and male mice are indicated with red (female) and blue (male) color. Black bars indicate genes that showed the most sex difference when its expression level is correlated with that of *Ifi202b*.

The following 18 genes were identified from the whole genome expression profiles of spleen in GeneNetwork of female and male mice: *Csf1r*, *FoxP3*, *Gata4*, *Ifnb1*, *Ifng*, *IL-10*, *IL-17a*, *IL-19*, *IL-20*, *IL-22*, *IL-22*, *IL-24*, *Nkx2-5*, *Tbx5*, *Tgfb1*, *Tgfb2*, *Tgfb3*, and *Tpr*. Probes for *Ifi200* family genes were identified from the database using key words “interferon inducible.” The degree of correlation of each of the 18 genes with the *Ifi200* family was analyzed. In male mice, the *Ifi200* family showed correlation among themselves; however, there was no correlation of the Ifi genes with any of these genes (Figure 2(a)). The Raa values for *Ifi202b*, *Ifi203*, *Ifi204*, and *Ifi205* to these 18 genes were 0.1189, 0.1799, 0.1306, and 0.144, respectively. In female mice, while *Ifi200* genes correlated with each other (Figure 2(b)), the Raa values for *Ifi202b*, *Ifi203*, *Ifi204*, and *Ifi205* to these 18 genes were 0.0825, 0.2107, 0.1510, and 0.1903, respectively. These values were slightly higher than those in male mice. Sex differences were compared using *R* values of each gene of the *Ifi200* family to the whole set of the 18 genes in this category. The *P* values for *Ifi202b*, *Ifi203*, *Ifi204*, and *Ifi205* to these 18 genes between female and male mice were 0.0004, 0.7425, 0.6775, and 0.1966, respectively. The *R* values between *Ifi202b* and these 18 genes between female and male mice were examined (Figure 2(c)). The result indicated that the correlation between the expression level of *Ifi202b* and most of the 18 genes (in particular, *Csf1r*, *Ifnb1*, *IL-20*, *IL-22*, *IL-24*, and *Tgfb2*) is stronger in male mice than those in female mice. In addition, both *Ifi204* and *Ifi205* were negatively correlated to *Gata4* (Figure 2(b)).

### 3.3. Sex Difference in Correlation with *Ifi200* Genes and the Lupus-Relevant Gene, *Jhdm1d*

**Figure 3:**
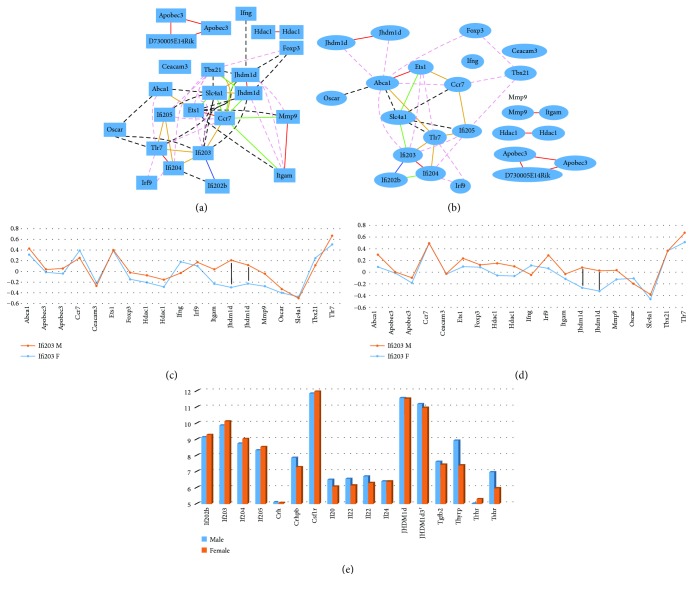
Gene network and sex difference between *Ifi200* family genes and reported lupus-relevant genes. (a) Gene network between *Ifi200* family genes and reported lupus-relevant genes in male mice. The 58 edges between the nodes, filtered from the 276 total edges and drawn as curves. (b) Gene network between *Ifi200* family genes and reported lupus-relevant genes in female mice. The 37 edges between the nodes, filtered from the 276 total edges and drawn as curves. In (c) and (d), the numbers on the vertical bar indicate the scale of *R* values between the expression level of *Ifi202b* and each gene listed at the bottom of the figure. Female and male mice are indicated with red (female) and blue (male) color. (c) Sex difference of correlation on expression levels between *Ifi203* and lupus-relevant genes in spleen. Black bars indicate that *Jhdm1d* showed most sex difference when its expression level is correlated with that of *Ifi203*. (d) Sex difference of correlation of expression levels between *Ifi205* and lupus-relevant genes in spleen. Black bars indicate that *Jhdm1d* showed the most sex difference when its expression level is correlated with that of *Ifi205*. (e) The expression levels of *Ifi200* family genes, some important immune-related genes, and Jhdm1d in female and male mice of strain NZB/BlNJ. The numbers on the vertical bar indicate the scale of the relative expression level of each gene. Gene names are listed at the bottom of the figure. Female and male mice are indicated with red (female) and blue (male) color.

Nineteen probes for 16 lupus-relevant genes (*Abca1*, *Apobec3*, *Ccr7*, *Ceacam3*, *Ets1*, *Foxp3*, *Hdac1*, *Ifng*, *Irf9*, *Itgam*, *Jhdm1d*, *Mmp9*, *Oscar*, *Slc4a1*, *Tbx21*, and *Tlr7*) were identified from the database of male murine spleen. Similar to that of *Ifi200*-relevant genes above, the correlation between the expression levels of these 17 genes and *Ifi200* genes was analyzed. Overall, the correlation in expression patterns between female and male mice was similar (Figures 3(a) and 3(b)). The *R* values between these 16 genes and the *Ifi200* gene family were 0.4921, 0.8198, 0.8788, and 0.8572, for *Ifi202b*, *Ifi203*, *Ifi204*, and *Ifi205*, respectively. In male spleen, the correlations among these 17 genes with *Ifi200* genes are stronger than those for the above 18 *Ifi200-*relevant immune important genes. The average absolute *R* values for *Ifi202b*, *Ifi203*, *Ifi204*, and *Ifi205* of these genes in male mice were 0.0725, 0.2621, 0.1752, and 0.1871, respectively. In female mice, the average *R* values were 0.103, 0.2058, 0.1474, and 0.1914, respectively. Interestingly, the expression levels of *Ifi203*, *Ifi204*, and *Ifi205* were all positively linked to that of *Tlr7* (Figures 3(a) and 3(b)) in both sexes. *P* values from *t*-test between female and male mice for the correlations between *Ifi200* genes and lupus-relevant genes were 0.4783, 0.0303, 0.4147, and 0.002 for *Ifi202b*, *Ifi203*, *Ifi204*, and *Ifi205*, respectively. Therefore, more detailed information between *Ifi203* and lupus-relevant genes and between *Ifi205* and genes in lupus pathway were obtained. As indicated in Figure 3(c), the correlation of *Ifi203* with *Jhdm1d* for female and male mice is different. *Jhdm1d* had two probes. The net sex differences of these two probes were 0.5 and 0.34. The net sex differences between *Ifi205* and two probes of Jhdm1d were 0.34 and 0.35. These data suggest that among genes in the lupus pathways, *Jhdm1d* may regulate the sex difference. Furthermore, we examined the expression levels of Ifi200 genes and genes of other important immune-related genes (shown in Figure 2) plus Jhdm1d in female and male mice of NZB/BlNJ which has been used for breeding of NZB/W [48]. The data shows that in the Ifi200 family, the expression levels of all four genes in female mice are higher than those in male mice. However, the expression levels of most of the immune-related genes and Jhdm1d in female mice are lower than those in male mice (except Csf1r and Trhr) (Figure 3(e)).

### 3.4. The Association between *Ifi200* Genes and Genes of OA and RA

**Figure 4:**
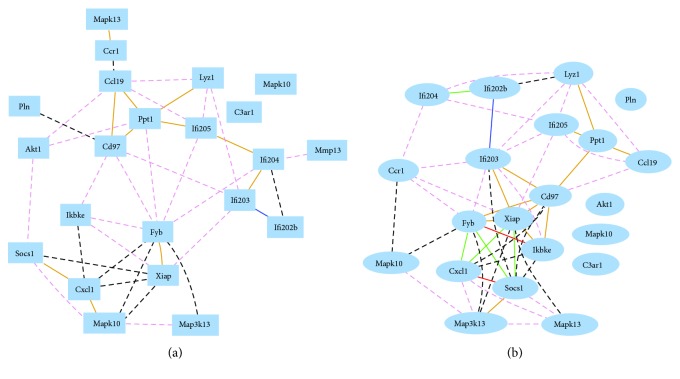
Gene network between genes of the *Ifi200* cluster and OA- and RA-relevant genes in mouse spleen. (a) Gene network between genes of the *Ifi200* cluster and OA- and RA-relevant genes in mouse spleen in male mice. The 22 nodes in the graph below show the selected traits. All nodes are displayed. The 41 edges between the nodes, filtered from the 231 total edges and drawn as curves. (b) Gene network between genes of the *Ifi200* cluster and OA- and RA-relevant genes in mouse spleen of male mice. The 21 nodes in the graph below show the selected traits. The 52 edges between the nodes, filtered from the 210 total edges and drawn as curves.

Twenty-one genes with relevance to OA and RA were identified from the whole genome expression profiles of spleen from female and male mice. These genes are *Akt1*, *C3ar1*, *Ccl19*, *Ccr1*, *Cd97*, *Cxcl1*, *Fyb*, *Ifi202b*, *Ifi203*, *Ifi204*, *Ifi205*, *Ikbke*, *Lyz1*, *Map3k13*, *Mapk10*, *Mapk10*, *Mapk13*, *Pln*, *Ppt1*, *Socs1*, and *Xiap*. In both female and male mice, the expression level of *Ifi205* showed a positive correlation with *Ppt1* (Figures 4(a) and 4(b)). The expression level of *Ifi203* showed positive correlation with *Cd97* and *Xiap* (Figures 4(a) and 4(b)), which confirmed a previous report [45, 46]. The probe for *Pln* was the only one that did not show any correlation in either male or female mice.

The average absolute Raa values for *Ifi202b*, *Ifi203*, *Ifi204*, and *Ifi205* for these genes in male mice were 0.1012, 0.2684, 0.1731, and 0.2482, respectively. In female mice, the average Raa values were 0.0847, 0.2061, 0.1418, and 0.2276. The *P* values between female and male mice for the *R* values between this set of OA- and RA-related genes and *Ifi202b*, *Ifi203*, *Ifi204*, and *Ifi205* were 0.1769, 0.2616, 0.0676, and 0.5872, respectively. The *R* values for these four groups between female and male mice were 0.5392, 0.7019, 0.4282, and 0.8063, respectively.

### 3.5. *Ifi202b* Showed Sex Difference in Correlation with Several Genes of Hormones

**Figure 5:**
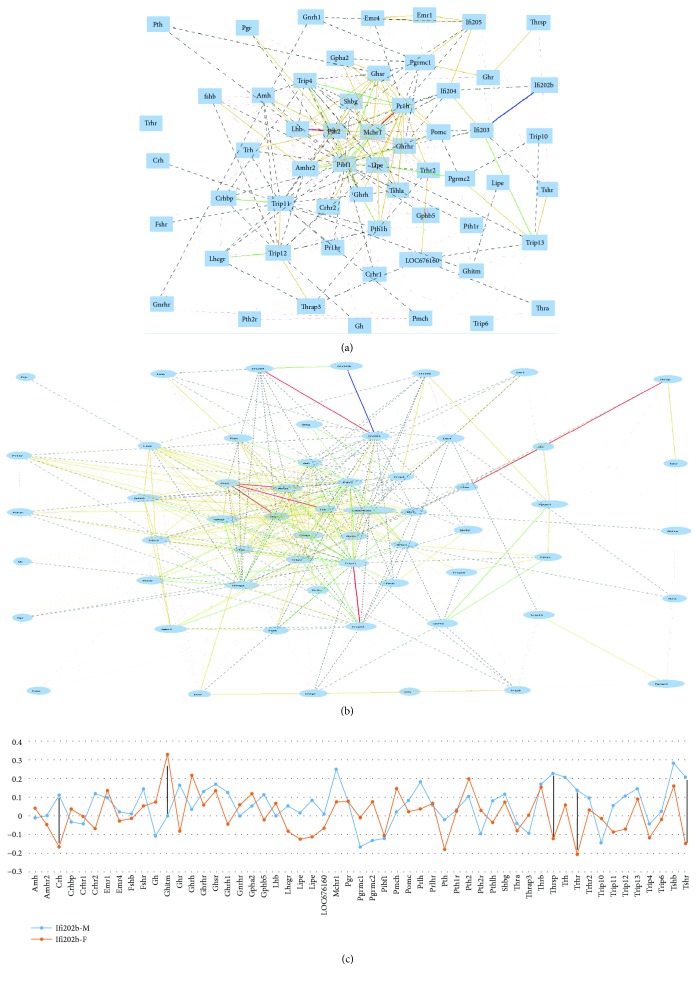
Gene network and sex difference between *Ifi200* family genes and sex hormone-related genes. (a) Gene network between *Ifi200* family genes and sex hormone-related genes in male mouse spleen. The 59 nodes in the graph below show the selected traits. The 312 edges between the nodes, filtered from the 1711 total edges and drawn as lines, show. (b) Gene network between the *Ifi200* family genes and sex hormone-related genes in female mouse spleen. The 59 nodes in the graph below show the selected traits. (c) Sex difference in correlation with expression levels between *Ifi202b* and sex hormone-related genes in spleen. The numbers on the vertical bar indicate the scale of *R* values between the expression level of *Ifi202b* and each gene listed at the bottom of the figure. Female and male mice are indicated with red (female) and blue (male) colors. Black bars indicate genes that show the most sex difference when its expression level is correlated with that of *Ifi202b*.

Probes for 55 sex hormone-related genes were identified by using key words “estradiol,” “progesterone,” and “testosterone” hormones. These genes were: *Amh*, *Amhr2*, *Crh*, *Crhbp*, *Crhr1*, *Crhr2*, *Emr1*, *Emr4*, *Fshb*, *Fshr*, *Gh*, *Ghitm*, *Ghr*, *Ghrh*, *Ghrhr*, *Ghsr*, *Gnrh1*, *Gnrhr*, *Gpha2*, *Gphb5*, *Lhb*, *Lhcgr*, *Lipe*, *Lipe*, *LOC676160*, *Mchr1*, *Pgr*, *Pgrmc1*, *Pgrmc2*, *Pibf1*, *Pmch*, *Pomc*, *Prlh*, *Prlhr*, *Pth*, *Pth1r*, *Pth2*, *Pth2r*, *Pthlh*, *Shbg*, *Thra*, *Thrap3*, *Thrb*, *Thrsp*, *Trh*, *Trhr*, *Trhr2*, *Trip10*, *Trip11*, *Trip12*, *Trip13*, *Trip4*, *Trip6*, *Tshb*, and *Tshr.* Their correlations with *Ifi200* family genes were analyzed (Figures 5(a) and 5(b)).

The average absolute Raa values for *Ifi202b*, *Ifi203*, *Ifi204*, and *Ifi205* with these genes in male mice were 0.0962, 0.1863, 0.1617, and 0.1761, respectively. In female mice, the average Raa values were 0.0567, 0.0820, 0.0945, and 0.12096. The *P* values between the sex hormone genes and *Ifi202b*, *Ifi203*, *Ifi204*, and *Ifi205* were 0.0243, 0.1329, 0.8053, and 0.4726, respectively. The *R* values for these four groups between female and male mice were, 0.0657, 0.6503, 0.6864, and 0.8131, respectively. The *P* value between gene sets of female and male mice was <0.05 and the *R* value was near 0.05. Associations between *Ifi202b* and sex hormone-related genes in female and male mice were further examined (Figure 5(c)). Correlations of several genes with *Ifi202b* showed gender dependence. The correlation between *Ifi202b* and *Crh* in male mice was 0.116 while in female mice, it was −0.165, with a net difference of 0.281. The correlation between *Ifi202b* and *Ghitm* in male mice was 0.002 while in female mice, it was 0.333, with a net difference of 0.331. The correlation between *Ifi202b* and *Thrsp* in male mice was 0.233 while in female mice it, was −0.121, with a net difference of 0.354. The correlation between *Ifi202b* and *Trhr* in male was 0.233 while in female, it was −0.121, with a net difference of 0.354. The correlation between *Ifi202b* and *Tshr* in male mice was 0.212 while in female mice it, was −0.145, with a net difference of 0.357. Thus, among the *Ifi200* family genes, *Ifi202b* plays a significant role in gender difference, in terms of interaction with sex hormone genes.

## 4. Discussion

Our data have revealed that the correlation expression levels of *Ifi200* genes with some immune-relevant genes have gender differences and suggest that different genes in the *Ifi200* family play different roles in male and female mice among different pathways of immune-mediated diseases. First, *Ifi2002b* showed the most sex difference in correlation with the 18 selected immune-relevant genes among all four *Ifi200* family genes. Second, *Ifi202b* showed gender difference in correlation with sex hormone genes [7]. Third, *Ifi203* and *Ifi205* showed significant gender difference in correlation with the genes in the lupus pathway. Fourth, none of the genes showed significant sex difference in correlation with genes selected for relevance to OA and RA. Our study also discovered key genes that potentially interact with *Ifi200* genes that are involved in regulating gender differences in pathological pathways of inflammatory and/or immune-mediated diseases.

Our initial analysis suggested that there was a potential gender difference in the expression level and regulation of *Ifi200* family genes. Our analysis obtained a *P* value of the *Ifi203* expression level of 0.0037 between female and male mice. Second, the QTL of *Ifi205* in female and male mice was mapped to different chromosomes. These differences provide a foundation for their potential sex differences in regulation of different immune pathways or diseases.

Our analyses suggest that there is sex difference between genes of the *Ifi200* family in their coexpression with some known coexpressed immune-related genes [28–32]. In particular, the coexpression of *Ifi202b* and these immune-related genes between female and male mice was significantly different with a *P* value of 0.0004. The correlation of expression levels between *Ifi202b* and six well-known, immune-related genes (*Csf1r*, *Ifnb1*, *IL-20*, *IL-22*, *IL-24*, and *Tgfb2)* is stronger in male mice than that in female mice. These genes are key components in the interleukin and *Tgf* pathways, which are essential for pathological processes in immune responses. In addition, both *Ifi204* and *Ifi205* were negatively correlated with *Gata4*. It has been reported that p204 (the protein product of *Ifi204*) is required for differentiation of P19 murine embryonal carcinoma cells to beating cardiac myocytes: its expression is activated by cardiac Gata4 and two other proteins, *Nkx2.5* and *Tbx5* [32].

These data suggest that, in the spleen, the expression levels of *Nkx2.5* and *Tbx5* may be influenced by *Ifi204* and *Ifi205* through Gata4.

Our data indicated that the gender difference in lupus disease might be caused by the molecular pathway that is regulated through interaction between *Ifi200* genes and genes in the lupus pathway. Among four genes, two of them, *Ifi203* and *Ifi205*, showed sex difference when correlated with the expression levels of genes in the lupus pathway. The correlation of both *Ifi203* and *Ifi205* to *Jhdm1d* between female and male mice is different. *Jhdm1d* had two probes. Both probes showed sex difference in the correlation of expression levels between two *Ifi200* genes and genes in the lupus pathway. Sex hormones have been known to moderate the susceptibility to lupus [7]. Our data strongly suggest that among genes in lupus pathways, *Jhdm1d* may regulate the sex difference through interaction with *Ifi200* genes. Surprisingly, we did not find significant sex difference between the correlation of expression levels of *Ifi200* genes and known OA- or RA-relevant genes. However, these data need to be confirmed in the future studies.

Our data also established a connection of gender differences with sex hormone genes. Sex hormone genes are regarded as key genes in the gender differences in diseases. *Ifi202b* plays a significant role in gender difference through interaction with sex hormones. The *P* values between the sex hormone genes and *Ifi202b* were 0.0243. Correlations of several genes to *Ifi202b* showed sex difference. These genes were *Crh*, *Ghitm*, *Thrsp*, *Trhr*, and *Tshr*. However, our comparisons did not show a significant gender difference between the rest of three genes in the *Ifi200* family and the sex hormone genes.

## 5. Conclusions

Four genes in the *Ifi200* family play different roles in sex difference among dissimilar pathways of these four gene groups. Different genes play different roles in sex difference in different diseases. In order to understand the molecular mechanism of sex difference in different immune diseases, it is essential to study the roles of genes of the *Ifi200* family.

## Data Availability

The data used for this study are at GeneNetwork (http://www.genenetwork.org/webqtl/main.py). These data are open to public and are freely available to readers.

## Abbreviations

AIM 2: Melanoma 2
BXD: C57BL/6J X DBA/2
eQTL: Expression quantitative trait loci
ifi: Interferon inducible
*Ifi200*: Interferon-inducible-200
IL: Interleukin
IP: Interferon-inducible protein
LRS: Likelihood ratio statistic
MNDA: Myeloid cell nuclear differentiation antigen
OA: Osteoarthritis
RA: Rheumatoid arthritis
RF: Rheumatoid factor
RI: Recombinant inbred
SAD: Spontaneous arthritis disease
SLE: Systemic lupus erythematosus
Tpr: Translocated promoter region.
